# Antidiarrhoeal Activity of the Alcoholic Extract of the Leaves of *Butea frondosa* Koen. Ex Roxb

**DOI:** 10.4103/0250-474X.65025

**Published:** 2010

**Authors:** D. Banji, Otilia Banji, M. Shanthmurthy, M. Singh

**Affiliations:** Nalanda College of Pharmacy, Charlapally, Hyderabad Road, Nalgonda-508 001, India; 1 Siddaganga College of Pharmacy, B. H. Road, Tumkur-572 102, India; 2 Balaji Institute of Pharmaceutical Sciences, Anantpur-515 001, India

**Keywords:** *Butea frondosa*, alcohol extract of the leaf, antidiarrhoeal, castor oil-induced diarrhea model

## Abstract

The study evaluated the antidiarrhoeal property of the alcohol extract of *Butea frondosa* leaf on mice and rats. Studies revealed that at a dose of 25 and 75 mg/kg a considerable reduction in the extent of diarrhoea was observed but at a dose of 100 mg/kg the animals appeared completely constipated when subjected to castor oil induced diarrhoea and intestinal motility model. Therefore, *Butea frondosa* can be regarded as an effective antidiarrhoeal.

Diarrhoea can be regarded as a global menace which can culminate in mortality and morbidity of the incumbent due to loss of fluids and electrolytes from the body. Diarrhoea, in fact, claims the lives of 5-8 million infants and children worldwide[1]. The plausible implicating factors inducing diarrhoea can range from infective to immunological and nutritive factors. Although nutritive supplements have proved beneficial in acute diarrhoea, chronic diarrhoea still remains elusive and can often culminate in serious effects if left untreated. In order to defend oneself from the onslaught of diarrhoea, it would be therefore worthwhile to use herbal-based products as the propensity of adverse effects with chemicals is high. *Butea frondosa* Koen. Ex Roxb (Papilionaceae) called the “Flame of the forest” has been traditionally used as an astringent, in colic, for worms and in piles[2]. *Butea frondosa* are bestowed with flavanoids, glucosides and lectins[3]. They are reported to possess nootropic and antistress properties[4]. Leaf extract is also capable of alleviating intraocular pressure[5] and inflammation[6]. In folklore medicine, *Butea frondosa* is being used as an antidiarrhoeal.

Although the stem bark extract of *Butea frondosa* was found to possess antidiarrhoeal activity, it would be important to suggest that every part of a plant is unique in function and is endowed with different proportion of active constituents and therefore might possess different therapeutic qualities. The rationality of using the leaves is more intense, as the leaf extract has been evaluated for its antiinflammatory action. Therefore we intended to explore the potential of the leaves of *Butea frondosa* to circumvent diarrhoea.

Fresh leaves of *Butea frondosa* was collected locally, authenticated by a Botanist and a voucher specimen bearing number B/SSCP/05 is preserved in the herbarium of our college. Leaves were cleaned, shade dried, powdered fine (40 g) and thoroughly extracted with ethanol using a Soxhlet extractor for 18 h. The leaf extract of *Butea frondosa* (LEBF) was filtered, concentrated and stored in a glass stoppered bottle at 4°. The desired quantity of the extract was suspended in 4% tragacanth and used.

For castor oil induced diarrhoea model, mice (20-30 g) from our own colony, housed under standardized animal house conditions were used. Food was withheld for 12 h prior to the experiment but water was provided *ad libitum*. Mice were randomly divided in to 5 groups comprising 6 animals each. The protocol was approved by the institutional animal ethical committee bearing no SSCP/15/2004-2005 dated 3/2/2005. Group-1 was treated with castor oil orally and served as the negative control, Group-2 was administered loperamide (1 mg/kg) and served as the positive control, Group-3 received extract containing 25 mg/kg LEBF, Group-4 received extract containing 75 mg/kg LEBF and Group-5 received extract containing 100 mg/kg. After 1 h of drug and vehicle treatment, all the groups were challenged with 1 ml of castor oil orally. Animals were observed for 4 h and the number of wet and dry droppings was counted every hour for a period of 4 h[7].

For gastrointestinal motility model, rats (180-200 g) from our own colony, housed under standardized animal house condition were divided into 5 groups consisting of 6 animals each. Animals were provided with water *ad libitum* and food was withheld from them for 18 h prior to the experiment. Group-1 was treated with 1 ml of 4% tragacanth orally and served as the negative control, Group-2 received loperamide (1 mg/kg) and served as the positive control, Group-3 was administered with 1ml extract containing 25 mg/kg LEBF orally, Group-4 received 1ml extract containing 75 mg/Kg LEBF orally and Group-5 received 1ml of the extract containing 100 mg/kg of LEBF. After 30 min, the intestinal motility was assessed by orally administering semisolid test charcoal meal consisting of 1 ml of deactivated charcoal (5% deactivated charcoal in 4% aqueous tragacanth). The animals were sacrificed 30 min later. The abdomen was opened and the entire small intestine starting from the pyloric end up to the ileocaecal end was removed and placed on blotting paper. The distance traveled by charcoal meal and the total length of the small intestine was measured in centimeters and expressed as percent intestinal transit[7]. The results are given as mean±SEM. Statistical analysis is done by unpaired Student t' test.

Table 1 elaborates the mean defecation and weight of droppings in mg/h for 4 h in the presence and absence of extract in the castor oil induced diarrheal test. The mean number of droppings recorded in the 1^st^, 2^nd^, 3^rd^ and 4^th^ h was 7±1.2, 9±1.26, 10±1.18 and 3±0.80, respectively. The corresponding weight of droppings was 210±3.26, 240±2.60, 250±2.10 and 132±2.60 mg, respectively. The findings reveal a statistical significant (p<0.05) reduction in the number and weight of droppings in groups receiving 25 mg/kg LEBF to 2±0.14 and 80±1.40 at 4 h compared to the control. Treatment with 75 mg/kg produced a further reduction in the number and weight of droppings. Groups that received 100 mg/kg of LEBF appeared completely constipated and therefore exhibited a statistically significant difference compared to the control (p<0.01). The percent intestinal transit as shown in Table 2 is reduced significantly with loperamide to 25.26±1.12. With doses of 25, 75 and 100 mg/kg of LEBF, the percent intestinal transit showed a significant reduction to 36.48±0.88, 27.43±2.10 and 11.69±0.29 compared to the control.

Castor oil induced diarrhoea and intestinal transit of contents are cardinal methods used to evaluate antidiarrhoeal activity in animals. Castor oil-induced diarrhoea is a suitable model, as it allows the observation of measurable changes in the number of stools, enteropooling and intestinal transit. Ricinoleic acid is generated by the action of lipase on castor oil, which is responsible for stimulating secretory processes, decreasing glucose absorption and primarily promoting motility of the small intestine[8]. Furthermore, ricinoleic acid from castor oil induces irritation and inflammation of the intestinal mucosa and this could be a triggering factor for the production of prostaglandins which enhances fluid and electrolytes in the small intestine[9]. It also activates the nitrinergic nervous system resulting in diarrhea[10]. Administration of a charcoal meal provides an insight into the extent of intestinal transit before and after drug administration. Agents which minimize the biosynthesis of prostaglandins could effectively reduce castor oil induced diarrhea. As data on the anti-inflammatory activity of LEBF is already available[6] it would be reasonable to point to the fact that LEBF could reduce the synthesis of prostaglandins, resulting in reduced motility of the GIT. *Butea frondosa* leaf extract at a dose of 100 mg/Kg has shown to exhibit significant anti-diarrhoeal propensity as the animals appear completely constipated. Flavanoids present as active principles in abundance in LEBF can be a contributing factor for its anti-diarrhoeal properties[11]. The ability of flavanoids to inhibit intestinal motility and block prostaglandin induced secretory process has been established[12]. The functioning of the gastrointestinal tract is largely regulated by the cholinergic, adrenergic and serotinergic neurohumoral systems[13]. Alterations in any of these enteric systems can serve as a powerful implicating factor in the induction of diarrhoea. Seed extract of *Butea frondosa* was found to oppose the action of acetylcholine and histamine on the guinea pig ileum[14]. Therefore, the delineation that the leaf extract of *Butea frondosa* may project the same action on these enteric neurotransmitters resulting in decreased propulsion of intestinal contents. This could therefore serve as another contributor to its anti-diarrhoeal action.

**Table 1:** ANTIDIARRHOEAL ACTIVITY OF LEAF EXTRACT OF *BUTEA FRONDOSA* IN CASTOR OIL-INDUCED DIARRHEA IN MICE

Groups	No. of animals	Dose	Mean number of droppings				Mean weight of droppings (mg)			
			1h	2h	3h	4h	1h	2h	3h	4h
Control (Castor oil)	05	1 ml	7±1.2	9±1.26	10±1.18	3±0.80	210±3.26	240±2.60	250±2.10	132±2.60
Loperamide	05	1 mg/kg	-	0.8±0.12*	1.21±0.22*	1.26±0.32*	135±0.56*	95±1.24*	86±1.86*	72±2.12*
LEBF+Castor oil	05	25 mg/kg + 1ml	5±0.98	4±0.78*	3±0.42*	2±0.14*	160±2.40*	150±3.10*	124±2.78*	80±1.40*
LEBF+Castor oil	05	75 mg/kg + 1 ml	3.2±0.46*	2.8±0.62*	2.0±0.16*	1.4±0.22*	148±2.15*	130.5±1.70*	101±1.66*	74±1.25*
LEBF+Castor oil	05	100 mg/kg + 1ml	‐‐	‐‐	‐‐	‐‐	‐‐	‐‐	‐‐	‐‐

Values are mean±SEM, leaf extract of *Butea frondosa* (LEBF),

* *P*<0.05 compared to control. At 100 mg/kg animals were completely constipated

**Table 2:** EFFECT OF THE LEAF EXTRACT OF *BUTEA FRONDOSA* ON SMALL INTESTINAL TRANSIT IN RATS.

Treatment	Total length of intestine (cm)	Distance traveled by charcoal meal (cm)	% intestinal transit
4% Tragacanth	70.21±0.32	35.13±0.48	50.03±1.02
Loperamide (1 mg/Kg)	69.78±0.12	18.11±0.80	25.95±1.12*
25 mg/Kg LEBF	69.76±0.44	25.45±0.69	36.48±1.88*
75 mg/Kg LEBF	69.85±1.24	19.16±0.94	27.43±2.10*
100 mg Kg LEBF	69.33±0.89	8.11±0.41	11.69±0.29*

Values are mean± SEM, N=5 observations in each group, leaf extract of *Butea frondosa*

* *P*<0.05 compared to control
